# Infants’ sensitivity to phonotactic regularities related to perceptually low-salient fricatives: a cross-linguistic study

**DOI:** 10.3389/fpsyg.2024.1367240

**Published:** 2024-03-06

**Authors:** Leonardo Piot, Thierry Nazzi, Natalie Boll-Avetisyan

**Affiliations:** ^1^Department of Linguistics, Cognitive Sciences, University of Potsdam, Potsdam, Germany; ^2^Integrative Neuroscience and Cognition Center, CNRS & Université Paris Cité, Paris, France

**Keywords:** phonotactics, salience, cross-linguistic, fricatives, infant language

## Abstract

**Introduction:**

Infants’ sensitivity to language-specific phonotactic regularities emerges between 6- and 9- months of age, and this sensitivity has been shown to impact other early processes such as wordform segmentation and word learning. However, the acquisition of phonotactic regularities involving perceptually low-salient phonemes (i.e., phoneme contrasts that are hard to discriminate at an early age), has rarely been studied and prior results show mixed findings. Here, we aimed to further assess infants’ acquisition of such regularities, by focusing on the low-salient contrast of /s/- and /ʃ/-initial consonant clusters.

**Methods:**

Using the headturn preference procedure, we assessed whether French- and German-learning 9-month-old infants are sensitive to language-specific regularities varying in frequency within and between the two languages (i.e., /st/ and /sp/ frequent in French, but infrequent in German, /ʃt/ and /ʃp/ frequent in German, but infrequent in French).

**Results:**

French-learning infants preferred the frequent over the infrequent phonotactic regularities, but the results for the German-learning infants were less clear.

**Discussion:**

These results suggest crosslinguistic acquisition patterns, although an exploratory direct comparison of the French- and German-learning groups was inconclusive, possibly linked to low statistical power to detect such differences. Nevertheless, our findings suggest that infants’ early phonotactic sensitivities extend to regularities involving perceptually low-salient phoneme contrasts at 9 months, and highlight the importance of conducting cross-linguistic research on such language-specific processes.

## Introduction

Infants’ acquisition of their language-specific phonological system occurs rapidly. Indeed, already in their first year of life, infants start to specialize in speech sounds – or phonemes – that are used in their native language at the lexical level (e.g., Werker and Tees, 1984; Kuhl et al., 1992). Another important phonological property acquired during that period is the language-specific phonotactic system, that is, the legal and probabilistic positioning and sequencing of speech sounds within the words of a given language. Previous studies have shown that infants begin to acquire their language-specific phonotactic system in the first year (e.g., Friederici and Wessels, 1993; Jusczyk et al., 1994; Gonzalez-Gomez and Nazzi, 2012). However, the acquisition of regularities clearly contrasting in phonotactic frequencies but composed of perceptually low-salient phonemes has rarely been studied in infancy, and never with a cross-linguistic approach that makes it possible to ascertain that the findings result from the acquisition of language-specific properties rather than some intrinsic characteristics of the words presented to the infants. Indeed, only two studies investigated early phonotactic sensitivities to low-salient fricative patterns, and found contrasting results: French-learning infants were sensitive to frequent regularities of fricatives (Gonzalez-Gomez and Nazzi, 2015), whereas the evidence is more mixed for English-learning infants (Henrikson et al., 2020). Given these prior results, more research is needed to understand whether language-specific regularities involving low-salient phonemes are acquired early in development, and whether this depends on the infants’ native language. This study aims to further investigate infants’ acquisition of their language-specific phonotactic system from a cross-linguistic perspective by asking if German-and French-learning 9-month-old infants have already acquired perceptually low-salient regularities of fricative-plosive word-initial clusters.

Friederici and Wessels (1993) and Jusczyk et al. (1994) set the stage for the investigation of early phonotactic acquisition. Using the headturn preference procedure (HPP, Kemler Nelson et al., 1995) to investigate language-specific phonotactic sensitivities within one language, they demonstrated that sensitivity to language-specific phonotactic patterns emerges between 6 and 9 months: Friederici and Wessels (1993) tested Dutch-learning infants’ preferences for nonwords including legal (e.g., /bref/, /murt/) versus illegal (e.g., /febr/, /rtum/) consonant clusters (henceforth CCs) in Dutch. Phonotactically legal CCs were attested and typical in their specific positions within Dutch words, whereas illegal CCs were clusters that could not appear in their specific positions within Dutch words. Nine-month-olds preferred listening to the nonwords containing legal CCs at onsets and offsets, whereas 4-and 6-month-olds showed no listening preference. In parallel, Jusczyk et al. (1994) investigated early sensitivity to phonotactic probabilities in English, presenting English-learning 6- and 9-month-olds with lists of monosyllabic nonwords with a consonant-vowel-consonant (CVC) structure that had high (e.g., riss, ghen, kazz) or low (e.g., yowdge, shawch, gushe) positional uni-and biphone probabilities in English. Infants at 9 but not 6 months listened significantly longer to the high than to the low probability lists of monosyllables. These two studies have been taken as evidence that knowledge of both language-specific phonotactic legality and phonotactic probability is acquired between 6 and 9 months.

Capitalizing on these early findings, subsequent studies showed that in their first year of life infants also acquire non-adjacent regularities (i.e., regularities between non-sequential phonemes) between consonants (Nazzi et al., 2009; Gonzalez-Gomez and Nazzi, 2012; Gonzalez-Gomez et al., 2014) and vowels^1^ (Altan et al., 2016; Gonzalez-Gomez et al., 2019). Moreover, it has been found that infants’ phonotactic knowledge supports other early linguistic processes, such as wordform segmentation (Mattys and Jusczyk, 2001; Gonzalez-Gomez and Nazzi, 2013) and word learning (Graf Estes et al., 2011; MacKenzie et al., 2012; Gonzalez-Gomez et al., 2013).

Most studies mentioned above [with the exception of Gonzalez-Gomez et al. (2014, 2019)] tested infants within one native language. Although infants’ performance was in line with the phonotactic properties of their native language, which might attest to acquisition of native properties, it remains possible that language-general acoustic or structural properties of the words led to the preferences found. One way to avoid such a confound is to test, with the same task and words, groups of infants acquiring different languages with different phonotactic properties, and establish that their phonotactic preferences differ and relate to the properties of each language. The first crosslinguistic study on phonotactics compared Spanish-and Catalan-learning monolingual 10-month-olds, and two groups of Spanish-Catalan bilingual 10-month-olds differing in their predominant language (Sebastián-Gallés and Bosch, 2002). Using HPP, infants were presented with lists of nonwords containing coda CCs that were legal (e.g., /birt/, /dort/) versus illegal in Catalan (e.g., /ketr/ /bepf/). Importantly, Spanish being a language which forbids CCs in coda position, the two types of nonwords were phonotactically illegal in Spanish. The Catalan-learning monolinguals and both groups of bilinguals, but not the Spanish-learning monolinguals, preferred the list with phonotactic patterns legal in Catalan. A second study compared French-and Japanese-learning infants’ acquisition of non-adjacent dependencies, showing that French-learning infants develop a preference for labial-coronal sequences between 6 and 10 months, while Japanese-learning infants develop a preference for coronal-labial sequences between 10 and 13 months, in line with the respective phonotactic properties of French and Japanese (Gonzalez-Gomez et al., 2014). Lastly, a study comparing Hungarian-and French-learning infants established the emergence of a preference for harmonic words between 10 and 13 months in Hungarian, a harmonic language, but no such preference at 13 months in French, a non-harmonic language (Gonzalez-Gomez et al., 2019). Taken together, these cross-linguistic studies demonstrate that infants’ phonotactic sensitivity is tied to experience and knowledge of native language(s), rather than the acoustic/phonetic properties of the words presented. Interestingly, cross-linguistic studies also showed that phonotactic knowledge applies top-down, such that infants’ ability to discriminate between words composed of specific phonotactic patterns differs depending on the phonotactic system of the language they are learning. Since Japanese does not allow CCs, adult speakers of Japanese tend to perceptually repair these by perceiving an illusory/u/ between the two consonants, whereas speakers of languages allowing CCs do not perceive this illusory vowel (see Dupoux et al., 2011). Cross-linguistic infant studies have shown that this effect emerges early in life: at 14 months, while French-and English-learning infants can clearly discriminate between word pairs in which one of the words contains a consonant cluster and the other one contains a/u/between the two consonants of the cluster (e.g., abna vs. abuna), Japanese-learning infants show a reduced or no ability to do so (Mazuka et al., 2011; but see also Kajikawa et al., 2006).

While early phonotactic sensitivity has been demonstrated for infants learning a number of different languages, meta-analytic evidence suggests that the age at which it emerges partly depends on the specific type of regularity investigated, and on the specific language infants are acquiring (Sundara et al., 2022). However, little is currently known about the factors modulating acquisition, as most previous studies investigating infants’ sensitivity to phonotactic regularities used many different regularities in each phonotactic condition (e.g., 8 different legal versus 8 different illegal CCs embedded in 224 different nonwords; Sebastián-Gallés and Bosch, 2002) rather than focusing on specific phonotactic contrasts (but see, e.g., Gonzalez-Gomez and Nazzi, 2015). Furthermore, in most studies, phonotactic regularities between conditions usually differ by more than one phoneme (e.g., /rt./versus/pf/) with at least one perceptually salient phoneme contrast, involving early acquired phonemes that infants can produce early in life (i.e., at the babbling stage): vowels, plosives, and nasals (de Boysson-Bardies, 1996; Morgan and Wren, 2018; Lorenzini and Nazzi, 2022). Accordingly, there are important gaps in our knowledge of the specific types of phonotactic regularities acquired in infancy. As a result, several factors likely to modulate acquisition have been posited, such as input properties (e.g., how much evidence in different languages supports a specific pattern), usefulness of phonotactic knowledge (e.g., how much phonotactic regularities inform other linguistic processes such as word-learning), type of regularity (e.g., adjacent vs. non-adjacent, or consonant vs. vowel dependencies), and perceptual salience/position of the regularities within the words (e.g., Bonatti et al., 2005; Zamuner, 2006; Sundara et al., 2022). Yet, no study has directly established the role of these factors. In the present study, we test the potential involvement of one such factor: perceptual salience.

To assess infants’ acquisition of phonotactic regularities related to perceptually low-salient phonemes, the present study focused on fricative consonants. The definition of salience is still not clear across studies: understanding why some phonemes and phoneme contrasts are more salient than others, and which acoustic properties are linked to perceptual salience, is still a matter of investigation (for discussions, see Cristia et al., 2011; Chládková and Paillereau, 2020). Here, we define perceptual salience as infants’ ability to discriminate between phonemes that contrast in one phonetic feature. Fricatives are a class of sounds that can be considered as low-salient given that previous studies have presented mixed findings with regard to infants’ ability to perceive and discriminate fricative contrasts, which taken together suggest that discrimination of such contrasts is difficult in infancy. A set of studies (Eilers and Minifie, 1975; Eilers, 1977) suggests that English-learning infants cannot discriminate between fricative pairs such as /s/−/z/ and /f/−/θ/, but can discriminate between /s/−/v/ and /s/−/ʃ/. Moreover, Nittrouer (2001) tested English-learning infants on their discrimination between the fricative contrast /s/−/ʃ/ and either the vowel contrast /a/−/u/ or the stop voicing contrast /t/−/d/. Out of 15 infants who could discriminate vowel quality (either /sa/−/su/ or /ʃa/−/ʃu/), only 6 could also discriminate /sa/−/ʃa/, while out of 8 infants who could distinguish a stop voicing contrast (/ta/−/da/), none discriminated /sa/−/ʃa/. Importantly, the acquisition and perception of fricatives categories (or more specifically the /s/ and /ʃ/ categories) seems to be tightly linked to infants’ ambient linguistic input: Cristia (2011) showed that fine-grained, subphonemic aspects of the acoustic realization of /s/ in caregivers’ speech predicts 6-to 14-month-old infants’ discrimination of this sound from /ʃ/, suggesting that learning based on acoustic cue distributions of /s/ and /ʃ/ in the infants’ surrounding environment drives the acquisition of such categories. The fact that fricative contrasts are difficult to perceive by infants is relevant to phonotactic acquisition because the ability to discriminate between two phonemes is often a necessary requirement for phonotactic acquisition involving those phonemes. This was suggested by Zamuner (2006), who showed that Dutch-learning 9- and 11-month-olds do not display knowledge of the phonotactic final devoicing rule (resulting in allowing voiceless but no voiced plosives at Dutch word endings), possibly because infants at 9, 11 and 16 months do not show an ability to discriminate the voicing contrast in that position either.

At present, the only two studies that have explored infants’ acquisition of phonotactic patterns related to fricatives have provided contrasting results. The first study (Gonzalez-Gomez and Nazzi, 2015) focused on the acquisition of non-adjacent dependencies linked to the relative order of labial and coronal consonants, asking whether infants’ knowledge of such phonotactic constraints can be found when testing them with different types of consonants. The rationale was that in French, labial-coronal structures are more frequent than coronal-labial structures if both consonants are plosives or nasals, but coronal-labial structures are more frequent than labial-coronal structures if both consonants are fricatives. French-learning 10-month-olds preferred the most frequent phonotactic structures in all cases, showing a labial-coronal preference for plosive and nasal sequences, and a coronal-labial preference for fricative sequences. With respect to fricatives, this established that at 10 months, these infants could discriminate place of articulation of the fricatives, and had learned phonotactic regularities on these fricatives, hence indicating phonotactic acquisition linked to these perceptually low-salient phonemes. In the other study (Henrikson et al., 2020), English-learning full-and pre-term infants aged 7 to 14 months were tested on their preferences for phonotactic regularities involving low-salient phonemes, namely fricatives and liquids: frequent /ʃr/ and /sl/ versus infrequent /ʃl/ and /sr/ in word-initial position. No significant effects were found for the pre-term infants. For the full-term infants, an analysis restricted to the 9-month-olds showed a significant preference for the fricative-liquid patterns with the higher phonotactic probability in their language. However, when all four age groups (7, 9, 11, and 14 months) were analyzed together, no significant preference, nor interaction with age, was found, strongly reducing the significance of the effect found at 9 months. Hence, the results of this second study at best provide weak evidence of infants’ preferences for frequent fricative-liquid patterns.

In this context of contrasting results between the two previous studies, the main goal of the present crosslinguistic study was to further investigate whether 9-month-old infants are sensitive to phonotactic regularities involving perceptually low-salient phonemes, namely fricatives. This was done by assessing whether French-and German-learning 9-month-old infants are sensitive to language-specific word-initial fricative-plosive regularities, specifically word-initial /s/− versus /ʃ/−plosive CCs. We chose to focus on such regularities with a cross-linguistic perspective for two reasons. First, we wanted a very clear contrast in phonotactics, meaning that the frequent regularities had to be very frequent in both their uniphone frequencies (i.e., frequencies of the word-initial fricative) and biphone frequencies (i.e., frequencies of the word-initial fricative-plosive cluster), whereas the infrequent ones had to be very infrequent in both uniphone and biphone frequencies. Word-initial fricative-and specifically fricative-plosive clusters are overall very frequent in these two languages, but the frequency of distribution of these two types of regularities contrasts between French and German: word-initial /s/ and /s/−plosive clusters are very frequent in French and very infrequent (or even illegal, but present in loanwords) in German, while word-initial /ʃ/ and /ʃ/−plosive clusters are very infrequent (or even illegal, but present in loanwords) in French and very frequent in German. Second, while being clearly contrasted at the phonotactic level, these two patterns are also composed of low-salient, perceptually very similar phonemes, differing only in place of articulation.

In the present study, we hypothesized that infants’ phonotactic sensitivities at 9 months would extend to sensitivities to highly frequent versus infrequent regularities involving low-salient phonemes. Thus, given the between-language distributional contrast of /s/− and /ʃ/−plosive regularities described above, we predicted opposite preferences between infants of the two language groups (namely, a preference for /s/−plosive compared to /ʃ/−plosive in the French-learning group, and the opposite preference in the German-learning group). Note that novelty preferences can also be found in such designs, but given that it has rarely been documented in studies investigating phonotactic sensitivities (see Sundara et al., 2022; Figures 2 and 6), we consider a preference for the frequent phonotactic regularity within a language to be the more likely outcome. If confirmed, these findings would provide strong additional evidence of infants’ acquisition by 9 months of language-specific phonotactic properties related to low-salient phonemes, adding to the evidence found in the two prior related studies each testing only one language group (Gonzalez-Gomez and Nazzi, 2015; Henrikson et al., 2020).

## Methods

### Ethical statement

Parents of all infant participants provided written informed consent prior to the experiment. Both the experimental protocol and consent procedure were according to the principles expressed in the Declaration of Helsinki and approved by the ethics committees of both Université Paris Cité (Nr. 2011-03) and University of Potsdam (Nr. 42_2023).

### Participants

A total of 48 9-month-old infants from monolingual French (*N* = 24, mean age = 9.6 months, range = [9.1–10.1]) and German-speaking (*N* = 24, mean age = 9.5 months, range = [9.1–9.9]) families were included in the analyses. Seventeen additional infants (French-learning: *N* = 5; German-learning: *N* = 12) were tested but their data was not included in the final sample, because they were fussy (10), caregivers interacted with them during testing (2), they had otitis media within 5 days before the experimental session (1) or there was a technical/experimenter error (4). Finally, infants were included in the final sample only if they managed to complete at least one entire block (out of two blocks) before the experiment was stopped (one block included 12 experimental trials and two familiarization trials, see procedure below). Among all the participants included, 4 out of 24 French-learning infants did not finish the experiment, completing 20, 22, 24, and 26 trials out of 28, respectively. All German-learning infants completed the entire experiment.

All infants included in the analyses were in good health, had been born full-term (36–41 weeks of gestation), and had no known hearing or vision impairments. They were considered monolinguals, with a daily exposure to a single language (either French or German) above 80% of total language exposure as assessed through parental estimates (French-learning infants: *M* = 89.6%, *SD* = 8.7; German-learning infants: *M* = 97.5%, *SD* = 5.9). Families were contacted via the two babylabs’ participant databases and received a small gift for participation (i.e., a colorful diploma with the picture of their infant). In Germany, caregivers were additionally compensated for their time and travel by a small fee.

### Materials

#### Stimuli

Our stimuli consisted of 144 unique CCVCV nonwords, half of them starting with /s/ and the other half starting with /ʃ/, followed by either a /t/ or a /p/, thus giving four possible word-initial consonant clusters (i.e., /st/, /sp/, /ʃt/, /ʃp/), our phonotactic patterns of interest. These clusters were followed by one of 36 unique *VCV* tails and distributed such that the same tails appeared in both conditions (s-initial: /st/, /sp/ & ʃ-initial: /ʃt/, /ʃp/). The *VCV* structure of the tails was as follows: the first vowel was one of a set of six selected vowels attested and highly frequent in both French and German (/a/, /i/, /o/, /u/, /e/ & /y/). The onset of the second syllable included a variety of obstruents and sonorants (/k/, /g/, /d/, /b/, /m/, /n/, and /r/) to have acoustic variation between nonwords. No stimuli violated the OCP-Place constraint regarding the non-adjacent consonants C2 and C3: when the phoneme /p/ was present in the word-initial consonant cluster, C3 was never /m/ or /b/. When the phoneme /t/was present, C3 was never /n/or/d/. Finally, the word-final vowel was /a/ in one half and /i/ in the other half of the stimuli because these two vowels have a relatively comparable phonotactic probability in word-final position in the two languages, which was not the case for the rest of the vowels (e.g., notably, 70% of word-final vowels in German are schwas). The complete stimulus list can be found in Appendix 1 in Supplementary material.

#### Phonotactic probability

/s/−plosive and /ʃ/−plosive clusters were selected because they differ in phonotactic probability between French and German: word initially, both /s/− and /s/−plosive clusters are very frequent in French but infrequent in German, while both /ʃ/− and /ʃ/−plosive clusters are very frequent in German but infrequent in French. Calculations of phonotactic probability were performed using four different phonemically transcribed lexical databases: two based on adult speech, and two on infant-directed speech (IDS). For German, we used the German lemma database of CELEX (Baayen et al., 1995), including 51,322 number of different lemmas, and a lexical database by Stärk et al. (2022) derived from various CHILDES corpora (MacWhinney, 2000) including 1,660 number of word types. Similarly, for French, we used the LEXIQUE database (New et al., 2004), including 47,342 number of lemmas, and a French lexical database of infant-directed speech, including 5,533 number of word types. As no lexical database was publicly available for IDS, we derived our French IDS database from a corpus of phonemically transcribed IDS utterances (Carbajal et al., 2018), from which we segmented all unique words and extracted their frequency of occurrence to create a lexical database similar to the German IDS database. Following previous research showing that adults’ phonotactic intuitions are better captured by type frequency measures (Denby et al., 2018), we used type frequency for all our phonotactic measures, meaning that the frequency of occurrence of a given word in the database was not taken into account. Because the tails were identical for /s/− and /ʃ/−initial nonwords, differences in phonotactic probability between experimental lists within a language were driven solely by the word-initial clusters in the nonwords.

As in Jusczyk et al. (1994), phonotactic probability was operationally defined based on two main measures, and calculated word-initially in the two languages (see Tables 1, 2).

**Table 1:** Frequency counts (and probability) of /s/ & /ʃ/ and /s/− & /ʃ/−consonant clusters in German and French (adult lexical databases), calculated in word-initial positions.

	Uniphone			Biphone	
	German	French		German	French
/s/	165 (0.0032)	3,875 (0.0832)	/st/	8 (0.0002)	296 (0.0068)
			/sp/	6 (0.0001)	174 (0.0040)
/ʃ/	4,478 (0.0873)	804 (0.0173)	/ʃt/	1,452 (0.0283)	11 (0.0003)
			/ʃp/	816 (0.0159)	2 (<0.0001)

**Table 2:** Frequency counts (and probability) of /s/ & /ʃ/ and /s/− & /ʃ/−consonant clusters in German and French (lexical databases based on IDS corpora), calculated in word-initial positions.

	Uniphone			Biphone	
	German	French		German	French
/s/	2 (0.0012)	411 (0.0748)	/st/	1 (0.0006)	18 (0.0035)
			/sp/	0 (0)	11 (0.0021)
/ʃ/	142 (0.0855)	139 (0.0253)	/ʃt/	44 (0.0266)	2 (0.0004)
			/ʃp/	26 (0.0157)	3 (0.0006)

(1) Positional uniphone probability (i.e., how often a given phoneme occurs in a specific position within a word).
(2) Positional biphone probability (i.e., the phoneme-to-phoneme co-occurrence probability in a specific position within a word).

We also computed the overall probability of encountering the phonemes /s/ and /ʃ/ in each of the two languages’ lexicons (see Table 3).

**Table 3:** Frequency counts (and probability) of the phonemes /s/ and /ʃ/in German and French (adult language and IDS) among all consonants (not positional).

	ADS		IDS	
	German	French	German	French
s	12,687 (0.0288)	17,915 (0.0579)	324 (0.036)	1,399 (0.053)
ʃ	11,975 (0.0272)	2,732 (0.0088)	254 (0.0283)	347 (0.013)

Results of the lexical statistics suggest that /s/ and /s/−initial CCs are more frequent than /ʃ/ and /ʃ/−initial CCs word-initially in French in ADS (Table 1) and IDS (Table 2). The opposite pattern is found in German: both /ʃ/ and /ʃ/−initial CCs are more frequent than both /s/ and /s/−initial CCs word-initially in ADS (Table 1) and IDS (Table 2). When calculating overall frequency of /s/ and /ʃ/, it can be seen that the former is more frequent than the latter in both languages, although this difference is much more marked in French than in German (Table 3).

#### Recordings and trial lists

We asked two different speakers, one monolingual German-native female and one monolingual French-native female, to pronounce all our experimental stimuli. To control for indexicality (e.g., voice characteristics), at the same time allowing us to assess if infants’ phonotactic sensitivities are robust to acoustic-phonetic variability, we presented both language-specific pronunciations to our participants, in two experimental blocks counterbalanced for order of presentation across infants. Note that one set of studies had shown that French-learning infants’ preferences for the phonotactic regularities of their native language were not impacted by the native language of the person recording the stimuli, which was either a French (Nazzi et al., 2009; Gonzalez-Gomez and Nazzi, 2012; Gonzalez-Gomez et al., 2013) or a Japanese (Gonzalez-Gomez et al., 2014) monolingual speaker.

The two speakers were instructed to read the stimuli in an IDS register. Their productions were recorded in the same sound-proof booth with the same technical equipment. Nonwords were then organized into 12 lists of 12 items each for each speaker. Six lists contained stimuli starting with the /s/−plosive clusters, and six lists contained stimuli starting with the /ʃ/−plosive clusters. The length of the lists was kept constant. In each list, items were presented with a silent interstimulus interval which varied in duration, so that each list would last exactly 18 s. The average intensity of the nonwords was normalized at 70 dB using PRAAT (Boersma, 2001). The average duration (ms) and pitch (Hz) of the nonwords can be found in Table 4. Overall, /s/−initial nonwords were longer than /ʃ/−initial nonwords in both German (Mean difference = 100 ms) and French (mean difference = 20 ms), the difference being more marked in German. In contrast, /ʃ/−initial nonwords were on average characterized by a higher pitch than /s/−initial nonwords, in both German (Mean difference = −12.60 Hz) and French (Mean difference = −7.28 Hz), the difference being more marked in German. The German stimuli followed a strong-weak stress pattern, which is typical for German, whereas the French stimuli were pronounced with even stress on all syllables, which is typical for French.

**Table 4:** Mean (SD) duration and pitch of nonwords separated by condition and pronunciation.

	German		French	
	s-initial	ʃ-initial	s-initial	ʃ-initial
Duration (ms)	779 (68)	676 (43)	461 (31)	441 (25)
Pitch (Hz)	262 (14)	274 (17)	272 (17)	280 (13)

### Procedure, apparatus, and design

For the experimental procedure, we used the HPP set-ups in the babylabs of Paris and Potsdam. During testing, caregivers were seated in a sound-attenuated testing booth with their infants on their lap, facing forward. Loudspeakers were mounted into the walls of the two side panels at about the level of the infants’ heads. There were three lights mounted on the walls: a small green light directly in front of the infants, and two small red lights on either side of the infants, close to the two speakers. A video camera was also connected from below the central light (i.e., in front of the infants) to a monitor in an adjacent control room where the experimenter was located.

The experiment took place as follows. Infants and their caregiver (s) were welcomed by an experimenter. Caregivers first completed the consent form and were explained how the experiment would take place. Then a caregiver entered the testing booth with the infant. Once they were seated, the experiment started. The caregiver, who was instructed not to interact with the infant during testing, wore headphones playing experimental stimuli overlaid over music to efficiently mask the test stimuli. The experimenter, who recorded the infant’s looking behavior via button presses, was also blind to the conditions of the study as no sound from the testing booth reached the control room. Each trial began by drawing the infant’s attention to the center by flashing the central light. Once achieved, the central light was turned off and one of the two side lights started flashing. Once the infant turned and looked at it, the stimulus began to play (and the side light kept flashing during the trial). The trial ended when the entire stimulus for that trial had been played (an entire trial list lasted maximally 18 s) or when the infant turned away for at least a continuous period of 2 s. Infants’ attention to the stimuli was measured based on their looking time toward the target side light on a given trial.

The experiment was made up of a total of 28 trials, divided into two blocks, with one block consisting of the stimuli pronounced by the French native speaker, and the other of the stimuli pronounced by the German native speaker. Each block started with two warm-up trials consisting of classical music, one on each side, and was followed by 12 test trials consisting of six trials with the nonwords starting with /s/−plosive and six trials with the nonwords starting with /ʃ/−plosive. During each trial, a unique list of 12 stimuli was played (e.g., list 1: /stika/, /stoga/, /spiki/, /spogi/, /steba/, /spedi/, /spuni/, /stuma/, /spyni/, /stara/, /styga/, /spari/). Trial type (/s/−plosive versus /ʃ/−plosive), pronunciation (German versus French) and side orders (left versus right) were pseudorandomized across participants. We created eight versions of the experiment such that half of the participants started the experiment with the French pronunciation block and the other half with the German pronunciation block. Furthermore, half of the participants started the experiment with an /s/−plosive trial, and the other half with a /ʃ/−plosive trial. Finally, for half of the participants the first trial was on the left side of the booth, and for the other half it was on the right side of the booth. There were never more than two consecutive trials on the same side and no more than two consecutive trials of the same phonotactic condition in a row.

### Data pre-processing and analysis

All analyses were conducted in R-studio. We used linear mixed-effect models using the function lmer of the R package *lme4* (Bates et al., 2009), and the package *lmerTest* (Kuznetsova et al., 2017) to obtain *p*-values. We conducted a nested linear mixed-effect model, with infants’ log-transformed looking times (hereafter LT, in seconds) as the dependent variable. Given the distribution of the data, LTs were log-transformed (as also recommended by Csibra et al., 2016). As fixed factors, we included native language (French vs. German) as between-participant nesting factor, phonotactics (/s/−initial vs. /ʃ/−initial) and pronunciation (French vs. German) in interaction, and block (block1 vs. block2) as within-participant factors. All factors were sum-contrasted for the model (i.e., effect coding: −1 vs. 1). Individual participant intercepts and by-participant random slopes for pronunciation were included in the random effects structure. Phonotactics was not added as a by-participant random slope because the model failed to converge when doing so. The full equation was as follows:


$$\begin{array}{c} \log\;\left(LT\right)\sim \mathrm{Native}\;\mathrm{Language}/\\ \;\left(\mathrm{Phonotactics}\times \left(\mathrm{Pronunciation}+\mathrm{Block}\right)\right)\\ \;+\left(1+\mathrm{Pronunciation}|\mathrm{Participant}\right)\text{.} \end{array}$$

Note that we initially discussed whether we should compute a model with all factors in full interaction or the present nested one. Ultimately, we chose to compute the nested model as our confirmatory model for two reasons: (1) we were underpowered to reliably test for such interactions and (2) because both familiarity and novelty effects can emerge from experiments testing infants’ preferences, the nested model provided the best possible way to assess infants’ sensitivity to phonotactics without being restricted to one specific direction of preference, while also informing about the direction of this sensitivity.

## Results

**Figure 1:**
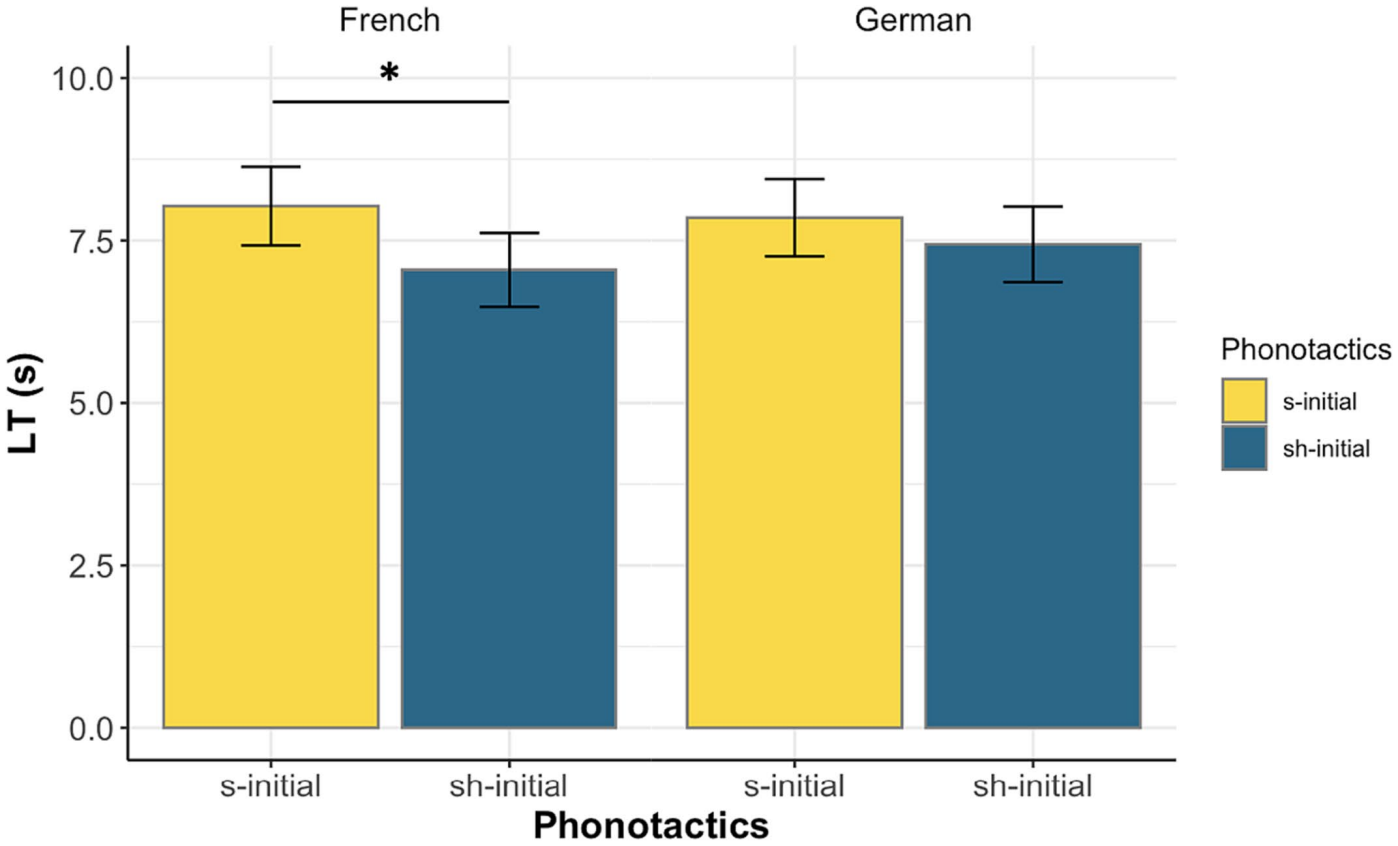
Raw infants’ LTs (Means and CIs), broken down by phonotactics and language group.

Eighteen out of 24 French-learning infants had longer LTs to the /s/−initial stimuli; 16 out of 24 German-learning infants had longer LTs to the /s/−initial stimuli. The results of the statistical model are presented in Table 5 and raw means and *CI*s are illustrated in Figure 1. Within the French-learning group, we found a significant main effect of phonotactics (*β* = −0.07, *SE* = 0.03, *p* = 0.032), indicating that infants’ LTs to the /s/−initial nonwords (*mean* = 7.99 s, *SD* = 1.90s) were longer than their LTs to the /ʃ/−initial nonwords (*mean* = 7.01 s, *SD* = 1.54 s). Within the German-learning group, the main effect of phonotactics was not significant (*β* = −0.03, *SE* = 0.031, *p* = 0.359) [s-initial: *mean* = 7.85 s, *SD* = 2.49 s; ʃ-initial: *mean* = 7.44 s, *SD* = 2.13 s]. There was also a significant main effect of block in both language groups (French: *β* = −0.23, *SE* = 0.03, *p* < 0.001; German: *β* = −0.09, *SE* = 0.03, *p* = 0.006), indicating that infants’ overall LTs decreased between the first and the second block. No further significant main effects or interactions were found.

**Table 5:** Results of the linear mixed model.

	log(LT)		
Predictors	Estimates	CI	*p*
(Intercept)	1.75	1.67–1.84	**<0.001**
Language	0.03	−0.05 – 0.12	0.442
Language [French]: Phonotactics	−0.07	−0.13 – −0.01	**0.031**
Language [German]: Phonotactics	−0.03	−0.09 – 0.03	0.359
Language [French]: Pronunciation	0.02	−0.04 – 0.08	0.564
Language [German]: Pronunciation	0.02	−0.04 – 0.09	0.447
Language [French]: block	−0.23	−0.29 – −0.17	**<0.001**
Language [German]: block	−0.09	−0.15 – −0.03	**0.006**
Language [French]:Phonotactics × Pronunciation	−0.04	−0.10 – 0.02	0.199
Language [German]: Phonotactics × Pronunciation	−0.04	−0.10 – 0.02	0.223
Language [French]:Phonotactics × block	−0.01	−0.07 – 0.05	0.764
Language [German]: Phonotactics × block	0.02	−0.04 – 0.08	0.585

*P*-values are highlighted in bold when smaller than 0.05.

## Discussion

In a cross-linguistic design, the present study investigated French-and German-learning infants’ sensitivity to low-salient, fricative-plosive word-initial phonotactic regularities at 9 months of age. To do so, we presented participants with lists of nonwords starting either with /s/−plosive clusters, with /s/ and /s/−plosive clusters being frequent word-initially in French but infrequent in German, or /ʃ/−plosive clusters, with /ʃ/ and /ʃ/−plosive clusters being frequent word-initially in German but infrequent in French, and measured their attention to either types of nonwords. Since previous studies found that infants start acquiring the phonotactics of their native language between 6 and 9 months (e.g., Friederici and Wessels, 1993), we expected our participants to show a significant preference for the frequent phonotactic regularities of their ambient language (French-learning participants: /s/−plosive clusters; German-learning participants: /ʃ/−plosive clusters). However, since the two previous studies on the acquisition of phonotactic properties related to perceptually low-salient fricatives found partly conflicting findings [with infants showing sensitivity in Gonzalez-Gomez and Nazzi (2015); but not at all ages in Henrikson et al. (2020)], it remained possible that infants would fail in the current experiment, or that performance would differ across languages. Our results show that the French-learning infants exhibited significantly longer LTs to the /s/−initial patterns, the more frequent regularities in their native language, than to the /ʃ/−initial patterns. In contrast, the German-learning group did not show a statistical preference for the frequent regularities in their native language.

These findings might be taken as evidence of cross-linguistic differences in infants’ phonotactic sensitivities, and in their trajectory of acquisition of phonotactic regularities. Results for the French-learning group are compatible with previous studies on phonotactic acquisition, and suggests that infants’ early phonotactic sensitivities extend to regularities involving perceptually low-salient, later-acquired phoneme contrasts in the French language. This is in line with the findings from Gonzalez-Gomez and Nazzi (2015) for another phonotactic regularity involving fricatives. Results for the German-learning group fail to provide evidence of knowledge of language-specific regularities on this low-salience fricative-based regularity. They contrast with the French results, but are in line with the difficulty found with English-learning infants tested on another fricative-based regularity in Henrikson et al. (2020).

To assess whether the difference in outcomes between our language-learning groups is statistically significant, we conducted an exploratory mixed effect model with a reduced number of parameters, with log (LT) as dependent variable and the two independent factors language and phonotactics in interaction (i.e., Log (LT) ~ Native Language x Phonotactics + (1|Participant)). This analysis only showed a main effect of phonotactics (*β* = −0.049, *SE* = 0.023, *p* = 0.034), indicating longer LTs to the /s/− than /ʃ/−plosive patterns, but failed to show a significant interaction between language and phonotactics (*β* = 0.02, *SE* = 0.023, *p* = 0.380). From this pattern of results, we cannot statistically conclude that the two groups of infants differed in their phonotactic sensitivities. To further assess if the data provides evidence for a null interaction or is just inconclusive, we used the Bayesian information criterion (BIC) for statistical inference, following Wagenmakers (2007). We computed two mixed effect models, one with and one without the interaction between language and phonotactics. We then extracted their respective BIC, and converted the BIC difference into a Bayes Factor, used to calculate the posterior probability of finding a null interaction (H0). This resulted in a Bayes Factor of 22.851, which amounts to a posterior probability of H0 of .96. This result can be interpreted as strong evidence that the data favors the null interaction, instead of being inconclusive (see Appendix 1 in Supplementary material for more details).

Based on this exploratory analysis, it is statistically more probable that both groups preferred the same phonotactic patterns (i.e., the /s/−plosive word-initial regularities), but the effect being smaller in the German-learning group, it did not emerge as significant in our confirmatory nested mixed-effect model. Another statistically less likely possibility, given that the data favors a null interaction between language and phonotactics, is that the two groups differed in their phonotactic sensitivities, with the French-learning group being sensitive to the frequent patterns in French and the German-learning group not being sensitive to the frequent pattern in German. However, the exploratory model was not sensitive enough to detect such a cross-linguistic difference with the current data. We discuss how we would interpret each of these two possibilities in what follows.

Let us consider first the possibility that both language groups do show a preference for the /s/−plosive word-initial regularity compared to the /ʃ/−plosive word-initial regularity. While this entails that both German-and French-learning infants are able to distinguish between the two low-salient phonemes /s/ and /ʃ/, results from the German-learning group are not in line with previous findings showing that infants prefer listening to the more frequent phonotactic regularities in their language. Since novelty phonotactic preferences have rarely been found (see Sundara et al., 2022), it seems unlikely that our findings could result from a cross-linguistic familiarity versus novelty preference.

An explanation for the overall preference for /s/−initial words could be found in the acoustic properties of our stimuli. Our /s/−initial nonwords were longer than our /ʃ/−initial nonwords, in both pronunciations (this difference being much more marked in the nonwords pronounced by the German native speaker). If 9-month-old infants are sensitive to durational differences in the range of 20-to 100-ms then the preference for /s/−plosive stimuli in both of our groups might partly be explained by such acoustic differences. Additionally, phonotactic sensitivity would reinforce French-but not German-learning infants’ preference for the /s/−plosive stimuli, which could explain the larger effect in French. Note that our stimuli also differed in pitch (with higher pitch for the /ʃ/−initial nonwords), which either did not affect performance, or contributed to the pattern observed if infants preferred the stimuli with lower pitch, an unlikely preference given data on IDS preference showing infants’ preference for stimuli with higher pitch (e.g., ManyBabies Consortium, 2020).

Finally, the general preference for the /s/− over /ʃ/−initial regularities might be linked to production factors. Productions studies suggest that /s/ is relatively easier to produce than /ʃ/, as illustrated by children experiencing a period of postalveolar fronting (the so-called “fis-effect,” e.g., pronouncing “fish” as “fis,” Jakobson, 1968; Kokkelmans, 2021), found in many languages including English (Vihman and Greenlee, 1987), German (Fox and Dodd, 1999), and French (Lemieux, 2011). Given studies showing that infants’ ability to produce consonants impacts their processing of speech between 9 to 11 months (DePaolis et al., 2011, 2013; Majorano et al., 2014), our perceptual preference for /s/ over /ʃ/ could relate to its production advantage. Note, however, that recent evidence from French-learning infants reports no production of /s/ or /ʃ/ in 32 11-month-olds, and only 1 infant producing /s/ and 1 producing /ʃ/ out of 32 14-month-olds (Lorenzini and Nazzi, 2022), so it is not clear that production of /s/ is favored at this developmental stage, and could have impacted their performance. One direct way to explore this possibility would have been to ask the caregivers about their infant’s babbling repertoire, and assess whether their production abilities are associated with their perceptual preferences in the current study. Since we did not collect this information, we leave this issue open for future research.

Let us now consider that our confirmatory nested mixed effect model shows cross-linguistic differences, with the French-learning group being sensitive to frequent regularities of low-salient phonemes in French while the German-learning group are not sensitive to frequent regularities of low-salient phonemes in German. What could explain this discrepancy between our two language-learning groups? Since French-learning infants were sensitive to our phonotactic manipulation, it seems unlikely that the lack of significant results in the German-learning group could be due to language-general processing abilities, such as for example an inability to discriminate fricatives at the age tested. Could the crosslinguistic difference be explained by phonotactic properties? This seems unlikely, since it was not the case that the difference in phonotactic frequency between conditions was more marked in French than in German. Indeed, both word-initial /ʃ/ and word-initial /ʃ/−plosive are comparatively much more common in German than word-initial /s/ and word-initial /s/−plosive are in French (see Tables 1, 2). This should have made it easier for German-than French-learning infants to acquire the respective phonotactic regularities. In contrast, our behavioral results might best be explained by sensitivity to overall/non-positional sound frequency within a language: while overall the phoneme /s/ is much more frequent than /ʃ/in French, the frequencies of these two phonemes are relatively similar in German (see Table 3). Thus, it might be that the preferences in our study are related to phoneme frequency rather than phonotactic frequency. This is reminiscent of another study investigating infants’ phonotactic preferences, which found that 7- but not 10-month-old French-learning infants prefer listening to coronal consonants compared to labial consonants, presumably because coronals are overall, as well as word-initially and word-finally, more frequent in French than labials (Gonzalez-Gomez and Nazzi, 2012). In our study, such sensitivity to non-positional phoneme frequencies is found at the intermediate age of 9 months, a possible delay related to the low-salience of the phonemes tested here. Further research will be needed on this finding, which was not predicted in the current study.

At any rate, our results further point toward the importance and the challenges of conducting cross-linguistic studies in language acquisition research, notably in the field of phonotactic acquisition. While in the current study, the frequency calculations would have predicted clear preferences in two opposite directions for our two language-learning groups, the lack of a significant result in the German-learning group suggests that frequencies are not enough to account for infants’ preferences at that age: it is possible that learners of different languages rely and process phonotactic information differently, possibly giving them different weights at different ages. Indeed, previous studies suggest that adult listeners’ use of specific phonotactic regularities for phoneme categorization differs depending on whether the adults were English or Dutch native-speakers, with English adults’ perception being affected to a greater extent by diphone probabilities than Dutch adults’ perception (Warner et al., 2005; Park et al., 2018; see Sundara et al., 2022 for a discussion). Relatedly, one cross-linguistic wellformedness judgment task points toward greater sensitivity to phonotactics in French compared to German adult listeners, although phonotactic probabilities predicted wellformedness judgments by both groups (Piot et al., 2024). It is possible that a differential use of phonotactic knowledge across individuals speaking different languages might already emerge in infancy and thus affect their sensitivities to phonotactic regularities. This is suggested by Jusczyk et al. (1993), documenting a similar discrepancy between English-and Dutch-learning infants, with stronger phonotactic sensitivity in English. For the specific case of fricatives tested here, findings for French suggest mastery of fricative-based properties by 9/10 months (Gonzalez-Gomez and Nazzi, 2015; current French data), while findings from English and German suggest failure or difficulties in acquiring fricative-based properties by the same age (Henrikson et al., 2020; current German data). The factors that drive these cross-linguistic differences (e.g., variable lexical stress, vocalic reduction, numerous complex codas and stress-timed rhythm in English/German, versus lack of lexical stress and vocalic reduction, less complex codas and syllable-timed rhythm in French) will have to be identified in future research.

Before concluding, we would like to point out some limitations of the present study. First, our experiment was relatively long for a 9-months-old infants’ preference study: it took between 7 to 10 min to complete. Experimental length, coupled with the large amount of information that our participants had to process (i.e., 144 different nonwords, pronounced by two speakers with different native languages), might have been cognitively too demanding for them, resulting in noisy data. It is possible that a shorter experiment, or a reduced number of different stimuli might have better suited our purposes. Nevertheless, additional exploratory analyses, for each experimental block separately, showed results that are similar, although not significant, to our main analysis: the main effect of phonotactics was marginally significant in both blocks for the French group (1st block: *p* = 0.108, 2nd block: *p* = 0.088), while it was not significant for the German group. The lack of significance for these exploratory analyses could relate to low statistical power, especially when considering only one block: although rather typical compared to similar studies on infants’ phonotactic acquisition, our sample size was relatively small. As a result, our effect would need to be further replicated, possibly with a bigger sample size. In any case, these explorations suggest that the relatively high number of trials was beneficial for detecting infants’ phonotactic preferences in our experiment.

In sum, our findings suggest that infants’ sensitivity to subtle, perceptually low-salient phonotactic patterns in their language at 9 months of age differs cross-linguistically. An implication for this finding is that infants’ early phonotactic knowledge is already detailed and fine-grained, at least in French-learners, while our German-learning infants failed to show a preference for the frequent phonotactic pattern in German. Further studies are needed to understand whether this cross-language discrepancy can be explained by overall phoneme frequency, the use of challenging phonological categories or differences between French and German.

## Data availability statement

The original contributions presented in the study are publicly available. The data and the R-script for data analysis are available on OSF: https://osf.io/pbk59/.

## Ethics statement

The studies involving humans were approved by Ethics Committees of both Université Paris Cité (Nr. 2011-03) and University of Potsdam (Nr. 42_2023). The studies were conducted in accordance with the local legislation and institutional requirements. Written informed consent for participation in this study was provided by the participants’ legal guardians/next of kin.

## Author contributions

LP: Conceptualization, Data curation, Formal analysis, Investigation, Methodology, Project administration, Visualization, Writing – original draft, Writing – review & editing. TN: Conceptualization, Formal analysis, Funding acquisition, Methodology, Resources, Supervision, Validation, Writing – review & editing. NB-A: Conceptualization, Formal analysis, Funding acquisition, Methodology, Resources, Supervision, Validation, Writing – review & editing.

## Footnotes

^1^Omane, P., Benders, T., and Boll-Avetisyan, N. (under review). Vowel harmony preferences in infants growing up in multilingual Ghana (Africa).

## References

[ref1] Altan A. Kaya U. Hohenberger A. (2016). Sensitivity of Turkish infants to vowel harmony in stem-suffix sequences: Preference shift from familiarity to novelty. In Proceedings of the 40th Boston University Conference on Language Development.

[ref2] Baayen R. H. Piepenbrock R. Gulikers L. (1995). The CELEX lexical database (release 2). Distributed by the linguistic data consortium, University of Pennsylvania.

[ref3] Bates D. Maechler M. Bolker B. Walker S. Christensen R. H. B. Singmann H.. (2009). Package ‘lme4’. Available at: https://lme4.r-forge.r-project.org.

[ref4] Boersma P. (2001). Praat, a system for doing phonetics by computer. Glot. Int. 5, 341–345.

[ref5] Bonatti L. L. Pena M. Nespor M. Mehler J. (2005). Linguistic constraints on statistical computations: the role of consonants and vowels in continuous speech processing. Psychol. Sci. 16, 451–459. doi: 10.1111/j.0956-7976.2005.01556.x 15943671

[ref6] Carbajal M. J. Bouchon C. Dupoux E. Peperkamp S. (2018). A toolbox for phonologizing French infant-directed speech corpora. IASCL Child Language Bulletin.

[ref7] Chládková K. Paillereau N. (2020). The what and when of universal perception: a review of early speech sound acquisition. Lang. Learn. 70, 1136–1182. doi: 10.1111/lang.12422

[ref8] Cristia A. (2011). Fine-grained variation in caregivers’/s/predicts their infants’/s/category. J. Acoust. Soc. Am. 129, 3271–3280. doi: 10.1121/1.3562562, PMID: 21568428 PMC3188616

[ref9] Cristia A. McGuire G. L. Seidl A. Francis A. L. (2011). Effects of the distribution of acoustic cues on infants' perception of sibilants. J. Phon. 39, 388–402. doi: 10.1016/j.wocn.2011.02.004, PMID: 21804656 PMC3145420

[ref10] Csibra G. Hernik M. Mascaro O. Tatone D. Lengyel M. (2016). Statistical treatment of looking-time data. Dev. Psychol. 52, 521–536. doi: 10.1037/dev0000083, PMID: 26845505 PMC4817233

[ref11] de Boysson-Bardies B. (1996). Comment la parole vient aux enfants Odile Jacob.

[ref12] Denby T. Schecter J. Arn S. Dimov S. Goldrick M. (2018). Contextual variability and exemplar strength in phonotactic learning. J. Exp. Psychol. Learn. Mem. Cogn. 44, 280–294. doi: 10.1037/xlm0000465, PMID: 28933893

[ref13] DePaolis R. Vihman M. M. Keren-Portnoy T. (2011). Do production patterns influence the processing of speech in prelinguistic infants? Infant Behav. Dev. 34, 590–601. doi: 10.1016/j.infbeh.2011.06.005, PMID: 21774986

[ref14] DePaolis R. Vihman M. M. Nakai S. (2013). The influence of babbling patterns on the processing of speech. Infant Behav. Dev. 36, 642–649. doi: 10.1016/j.infbeh.2013.06.007 23911593

[ref15] Dupoux E. Parlato E. Frota S. Hirose Y. Peperkamp S. (2011). Where do illusory vowels come from? J. Mem. Lang. 64, 199–210. doi: 10.1016/j.jml.2010.12.004

[ref16] Eilers R. E. (1977). Context-sensitive perception of naturally produced stop and fricative consonants by infants. J. Acoust. Soc. Am. 61, 1321–1336. doi: 10.1121/1.381435, PMID: 889607

[ref17] Eilers R. E. Minifie F. D. (1975). Fricative discrimination in early infancy. J. Speech Hear. Res. 18, 158–167. doi: 10.1044/jshr.1801.158, PMID: 1168827

[ref18] Fox A. V. Dodd B. J. (1999). ‘The phonological acquisition of German’. Sprache Stimme Gehoer.

[ref19] Friederici A. D. Wessels J. M. (1993). Phonotactic knowledge of word boundaries and its use in infant speech perception. Percept. Psychophys. 54, 287–295. doi: 10.3758/BF03205263, PMID: 8414887

[ref20] Gonzalez-Gomez N. Hayashi A. Tsuji S. Mazuka R. Nazzi T. (2014). The role of the input on the development of the LC bias: a crosslinguistic comparison. Cognition 132, 301–311. doi: 10.1016/j.cognition.2014.04.004, PMID: 24858107

[ref21] Gonzalez-Gomez N. Nazzi T. (2012). Acquisition of nonadjacent phonological dependencies in the native language during the first year of life. Infancy 17, 498–524. doi: 10.1111/j.1532-7078.2011.00104.x, PMID: 32693545

[ref22] Gonzalez-Gomez N. Nazzi T. (2013). Effects of prior phonotactic knowledge on infant word segmentation: the case of nonadjacent dependencies. J. Speech Lang. Hear. Res. 56, 840–849. doi: 10.1044/1092-4388(2012/12-0138), PMID: 23275409

[ref23] Gonzalez-Gomez N. Nazzi T. (2015). Constraints on statistical computations at 10 months of age: the use of phonological features. Dev. Sci. 18, 864–876. doi: 10.1111/desc.12279, PMID: 25530121

[ref24] Gonzalez-Gomez N. Poltrock S. Nazzi T. (2013). A “bat” is easier to learn than a “tab”: effects of relative phonotactic frequency on infant word learning. PLoS One 8:e59601. doi: 10.1371/journal.pone.0059601, PMID: 23527227 PMC3603888

[ref25] Gonzalez-Gomez N. Schmandt S. Fazekas J. Nazzi T. Gervain J. (2019). Infants’ sensitivity to nonadjacent vowel dependencies: the case of vowel harmony in Hungarian. J. Exp. Child Psychol. 178, 170–183. doi: 10.1016/j.jecp.2018.08.014, PMID: 30380456

[ref26] Graf Estes K. Edwards J. Saffran J. R. (2011). Phonotactic constraints on infant word learning. Infancy 16, 180–197. doi: 10.1111/j.1532-7078.2010.00046.x, PMID: 21297877 PMC3032547

[ref27] Henrikson B. Seidl A. Soderstrom M. (2020). Perception of sibilant–liquid phonotactic frequency in full-term and preterm infants. J. Child Lang. 47, 893–907. doi: 10.1017/S0305000919000825, PMID: 31852556

[ref28] Jakobson R. O. (1968). “Child language: aphasia and phonological universals” in Of Janua Linguarum, series minor. ed. Keiler A. R. (Berlin, Boston: De Gruyter Mouton)

[ref29] Jusczyk P. W. Friederici A. D. Wessels J. M. Svenkerud V. Y. Jusczyk A. M. (1993). Infants′ sensitivity to the sound patterns of native language words. J. Mem. Lang. 32, 402–420. doi: 10.1006/jmla.1993.1022

[ref30] Jusczyk P. W. Luce P. A. Charles-Luce J. (1994). Infants′ sensitivity to phonotactic patterns in the native language. J. Mem. Lang. 33, 630–645. doi: 10.1006/jmla.1994.1030

[ref31] Kajikawa S. Fais L. Mugitani R. Werker J. F. Amano S. (2006). Cross-language sensitivity to phonotactic patterns in infants. J. Acoust. Soc. Am. 120, 2278–2284. doi: 10.1121/1.2338285, PMID: 17069323

[ref46] Kemler Nelson D. G. K. Jusczyk P. W. Mandel D. R. Myers J. Turk A. Gerken L. (1995). The head-turn preference procedure for testing auditory perception. Infant Behav. Dev. 18, 111–116. doi: 10.1016/0163-6383(95)90012-8

[ref32] Kokkelmans J. (2021). The phonetics and phonology of sibilants: A synchronic and diachronic OT typology of sibilant inventories. Doctoral thesis. Italy: University of Verona.

[ref33] Kuhl P. K. Williams K. A. Lacerda F. Stevens K. N. Lindblom B. (1992). Linguistic experience alters phonetic perception in infants by 6 months of age. Science 255, 606–608. doi: 10.1126/science.1736364, PMID: 1736364

[ref34] Kuznetsova A. Brockhoff P. B. Christensen R. H. B. (2017). lmerTest package: tests in linear mixed effects models. J. Stat. Softw. 82. 1–26. doi: 10.18637/jss.v082.i13

[ref35] Lemieux G. (2011). ‘Le développement de la prononciation’. Online document.

[ref36] Lorenzini I. Nazzi T. (2022). Early recognition of familiar word-forms as a function of production skills. Front. Psychol. 13:947245. doi: 10.3389/fpsyg.2022.947245, PMID: 36186391 PMC9524451

[ref37] MacKenzie H. K. Curtin S. Graham S. A. (2012). 12-month-olds' phonotactic knowledge guides their word-object mappings. Child Dev. 83, 1129–1136. doi: 10.1111/j.1467-8624.2012.01764.x, PMID: 22537246

[ref38] MacWhinney B. (2000). The CHILDES project: Tools for analyzing talk. Third Edition. Mahwah, NJ: Lawrence Erlbaum Associates.

[ref39] Majorano M. Vihman M. M. DePaolis R. A. (2014). The relationship between infants’ production experience and their processing of speech. Lang. Learn. Dev. 10, 179–204. doi: 10.1080/15475441.2013.829740

[ref40] ManyBabies Consortium (2020). Quantifying sources of variability in infancy research using the infant-directed-speech preference. Adv. Methods Pract. Psychol. Sci. 3, 24–52. doi: 10.1177/2515245919900809

[ref41] Mattys S. L. Jusczyk P. W. (2001). Phonotactic cues for segmentation of fluent speech by infants. Cognition 78, 91–121. doi: 10.1016/S0010-0277(00)00109-8, PMID: 11074247

[ref42] Mazuka R. Cao Y. Dupoux E. Christophe A. (2011). The development of a phonological illusion: a cross-linguistic study with Japanese and French infants. Dev. Sci. 14, 693–699. doi: 10.1111/j.1467-7687.2010.01015.x, PMID: 21676090

[ref43] Morgan L. Wren Y. E. (2018). A systematic review of the literature on early vocalizations and babbling patterns in young children. Commun. Disord. Q. 40, 3–14. doi: 10.1177/1525740118760215

[ref45] Nazzi T. Bertoncini J. Bijeljac-Babic R. (2009). A perceptual equivalent of the labial-coronal effect in the first year of life. J. Acoust. Soc. Am. 126, 1440–1446. doi: 10.1121/1.3158931, PMID: 19739757

[ref47] New B. Pallier C. Brysbaert M. Ferrand L. (2004). Lexique 2: a New French lexical database. Behav. Res. Methods Instrum. Comput. 36, 516–524. doi: 10.3758/bf03195598, PMID: 15641440

[ref48] Nittrouer S. (2001). Challenging the notion of innate phonetic boundaries. J. Acoust. Soc. Am. 110, 1598–1605. doi: 10.1121/1.1379078, PMID: 11572369

[ref50] Park S. Hoffmann M. Shin P. Z. Warner N. L. (2018). The role of segment probability in perception of speech sounds. J. Acoust. Soc. Am. 143:1920. doi: 10.1121/1.5036263

[ref51] Piot L. Nazzi T. Boll-Avetisyan N. Auditory phonotactic wellformedness intuitions depend on the nativeness of a speaker’s pronunciation. Abstract, Linguistic Evidence (2024).

[ref52] Sebastián-Gallés N. Bosch L. (2002). Building phonotactic knowledge in bilinguals: role of early exposure. J. Exp. Psychol. Hum. Percept. Perform. 28, 974–989. doi: 10.1037/0096-1523.28.4.974, PMID: 12190262

[ref54] Stärk K. Kidd E. Frost R. L. (2022). Word segmentation cues in German child-directed speech: a corpus analysis. Lang. Speech 65, 3–27. doi: 10.1177/0023830920979016, PMID: 33517856 PMC8886305

[ref55] Sundara M. Zhou Z. L. Breiss C. Katsuda H. Steffman J. (2022). Infants' developing sensitivity to native language phonotactics: a meta-analysis. Cognition 221:104993. doi: 10.1016/j.cognition.2021.104993, PMID: 34953268

[ref56] Vihman M. M. Greenlee M. (1987). Individual differences in phonological development: ages one and three years. J. Speech Hear. Res. 30, 503–521. doi: 10.1044/jshr.3004.503 3695444

[ref57] Wagenmakers E. J. (2007). A practical solution to the pervasive problems of p values. Psychon. Bull. Rev. 14, 779–804. doi: 10.3758/BF03194105, PMID: 18087943

[ref58] Warner N. Smits R. McQueen J. M. Cutler A. (2005). Phonological and frequency effects on timing of speech perception: a database of Dutch diphone perception. Speech Comm. 46, 53–72. doi: 10.1016/j.specom.2005.01.003

[ref59] Werker J. F. Tees R. C. (1984). Cross-language speech perception: evidence for perceptual reorganization during the first year of life. Infant Behav. Dev. 7, 49–63. doi: 10.1016/S0163-6383(84)80022-3

[ref60] Zamuner T. S. (2006). Sensitivity to word-final phonotactics in 9-to 16-month-old infants. Infancy 10, 77–95. doi: 10.1207/s15327078in1001_5, PMID: 33412672

