# Supplementary material for: Infants’ sensitivity to phonotactic regularities related to perceptually low-salient fricatives: a cross-linguistic study
Source: Front Psychol. 2024 Mar 6;15:1367240. doi: 10.3389/fpsyg.2024.1367240 (PMC10964922; doi:10.3389/fpsyg.2024.1367240)
Supplement: Supplementary file 1 [file Data_Sheet_1.docx]

Appendix 1: complete set of experimental stimuli:

list 1:  /stika/, /stoga/, /spiki/, /spogi/, /steba/, /spedi/, /spuni/, /stuma/, /spyni/, /stara/, /styga/, /spari/

list 2: /stoka/, /stega/, /spoki/, /spegi/, /stuba/, /spudi/, /spani/, /stama/, /spydi/, /stira/, /styba/, /spiri/

list 3: /steka/, /stuga/, /staba/, /stima/, /stora/, /speki/, /spugi/, /spadi/, /spini/, /spori/, /styma/, /spygi/

list 4: /spaka/, /spiga/, /spoda/, /spena/, /spura/, /staki/, /stigi/, /stobi/, /stemi/, /sturi/, /spyka/, /styri/

list 5: /spika/, /spoga/, /speda/, /spuna/, /spara/, /stiki/, /stogi/, /stebi/, /stumi/, /stari/, /spyga/, /stymi/

list 6: /spoka/, /spega/, /spuda/, /spana/, /spira/, /stoki/, /stegi/, /stubi/, /stami/, /stiri/, /spyda/, /stybi/

list 7:  /ʃtika/, /ʃtoga/, /ʃpiki/, /ʃpogi/, /ʃteba/, /ʃpedi/, /ʃpuni/, /ʃtuma/, /ʃpyni/, /ʃtara/, /ʃtyga/, /ʃpari/

list 8: /ʃtoka/, /ʃtega/, /ʃpoki/, /ʃpegi/, /ʃtuba/, /ʃpudi/, /ʃpani/, /ʃtama/, /ʃpydi/, /ʃtira/, /ʃtyba/, /ʃpiri/

list 9: /ʃteka/, /ʃtuga/, /ʃtaba/, /ʃtima/, /ʃtora/, /ʃpeki/, /ʃpugi/, /ʃpadi/, /ʃpini/, /ʃpori/, /ʃtyma/, /ʃpygi/

list 10: /ʃpaka/, /ʃpiga/, /ʃpoda/, /ʃpena/, /ʃpura/, /ʃtaki/, /ʃtigi/, /ʃtobi/, /ʃtemi/, /ʃturi/, /ʃpyka/, /ʃtyri/

list 11: /ʃpika/, /ʃpoga/, /ʃpeda/, /ʃpuna/, /ʃpara/, /ʃtiki/, /ʃtogi/, /ʃtebi/, /ʃtumi/, /ʃtari/, /ʃpyga/, /ʃtymi/

list 12: /ʃpoka/, /ʃpega/, /ʃpuda/, /ʃpana/, /ʃpira/, /ʃtoki/, /ʃtegi/, /ʃtubi/, /ʃtami/, /ʃtiri/, /ʃpyda/, /ʃtybi/

Appendix 2: Computing Bayes Factor (exploratory analysis)

We followed Wagenmakers (2007), resulting in the following steps:

1. Extract BIC from two different mixed effect models, one with the interaction between language and phonotactics and the other without the interaction:

- Model 1 (interaction): log(LT) ~ language * Phonotactic +(1 | participant):

**BIC1 = 2729.939**

- Model 2 (no interaction) : log(LT) ~ language + Phonotactic +(1| participant):

**BIC0 = 2723.681**

1. Calculate Bayes Factor:

- BF01 = exp((BIC1 - BIC0)/2) = exp(6.258/2) = exp(3.129) ≈ **22.851**

With equal priors on the models, this would amount to a posterior probability of H0 of 22.851 / 23.851 ≈ **.96**, which can be interpreted as strong evidence that the data favors the null interaction, instead of being inconclusive.
